# High-dose glucocorticoids: improving outcomes of emergency laparotomy by reducing inflammation

**DOI:** 10.1093/bjs/znae153

**Published:** 2024-07-19

**Authors:** Dileep N Lobo

The metabolic response to injury and surgical trauma has developed teleologically to help preserve life during intervals of metabolic stress. It is a neuro-immuno-humoral response that helps maintain intravascular volume by the retention of salt and water, causes stress hyperglycaemia to provide substrate for metabolism and energy generation, and helps combat infection and promote wound healing. However, when there is no intervention and the response is allowed to continue unabated, it can cause harm via an inflammatory cascade that leads to increased vascular permeability, damage to the endothelial glycocalyx, and altered cellular, muscle, and organ metabolism that lead to circulatory shock, decreased tissue perfusion, organ dysfunction, and eventually death. The extent of the response is proportional to the magnitude of the injury and is amplified in the presence of pre-existing inflammation. The premise of Enhanced Recovery After Surgery (ERAS) pathways is to attenuate this stress response with the help of multimodal interventions in the perioperative interval that lead to accelerated recovery from surgery, fewer complications, and reduced length of hospital stay^1^.

Administration of glucocorticoids is a means of reducing perioperative inflammation and low-dose glucocorticoids have been used before surgery and intraoperatively to reduce postoperative nausea and vomiting without an increase in adverse events^2^. Glucocorticoids may also improve survival and reduce disease recurrence after major oncological surgery^3^.

In this issue of *BJS*, Cihoric *et al*.^4^ have reported the results of an RCT in which they gave patients undergoing emergency laparotomy 1 mg/kg dexamethasone before surgery to help attenuate the inflammatory response and, perhaps, improve outcomes when compared with the placebo. A dose of 1 mg/kg dexamethasone is the equivalent of 26.7 mg/kg hydrocortisone, 6.7 mg/kg prednisolone, and 5.3 mg/kg methylprednisolone (https://www.mdcalc.com/calc/2040/steroid-conversion-calculator) and would amount to 1866.7 mg hydrocortisone in a person weighing 70 kg and 2666.7 mg in a person weighing 100 kg.

A single high dose of dexamethasone has been used previously in cardiac surgery in an RCT of nearly 5000 participants^5^. The primary endpoint of that study was a composite of death, myocardial infarction, stroke, renal failure, or respiratory failure within 30 days of surgery and was not significantly different between the dexamethasone and placebo groups. However, the duration of postoperative mechanical ventilation, length of ICU stay, and length of hospital stay were significantly shorter in the dexamethasone group. Higher blood glucose concentrations were found in the group that received dexamethasone^5^. In addition, another RCT comparing high-dose preoperative methylprednisolone (10 mg/kg; the equivalent of 1.9 mg/kg dexamethasone) with 8 mg dexamethasone in 174 participants undergoing liver surgery showed no difference in the occurrence of postoperative complications^6^.

The sample size calculation for the trial of Cihoric *et al*.^4^ was based on the primary endpoint of reducing serum C-reactive protein (CRP) concentrations and the investigators were able to demonstrate significantly lower CRP concentrations in participants who received dexamethasone overall and also in the subgroups with bowel obstruction and peritonitis when compared with those who did not receive dexamethasone. This effect lasted until postoperative day 3 and by day 5 there were no differences between the groups. This clearly shows that high-dose dexamethasone had an appreciable effect on reducing inflammation. Although the study was not powered for the secondary endpoints, participants who received dexamethasone also demonstrated better haemodynamic parameters, less fluid requirements and overload, better peak expiratory flow rate, less fatigue, and earlier mobilization than those who received the placebo. Major postoperative complications were reduced in the dexamethasone group and, although there was no statistically significant difference in mortality at 30 days, the relative risk of dying at 90 days was 77% less in the dexamethasone group.

So, why did the results of this trial differ from those of previously published similar works? It was performed in the emergency setting and the participants, who had either bowel obstruction or peritonitis, had a significant amount of background inflammation, which was compounded by the stress of surgery. Perhaps, in the presence of background inflammation, the effects of dexamethasone were more pronounced. In addition, patients without postoperative complications have previously been shown to have better long-term survival than those with major complications^7^. A reduction in the degree of postoperative inflammation could have diminished the increase in capillary permeability, resulting in participants in the dexamethasone group requiring less intravenous fluid and developing less cumulative positive fluid balance, both of which have a bearing on outcome. Reduced inflammation may also have led to less fatigue, earlier mobilization, and less pain during mobilization. It is also important to note that both groups were managed with a well-defined and established ERAS pathway^1^.

Concerns about the use of low- and high-dose glucocorticoids include neuropsychiatric complications^8^ and the development of hyperglycaemia^5,9,10^. Blood glucose concentrations were significantly higher in the dexamethasone group than in the placebo group at 6 h after surgery and on postoperative days 1 and 2, with no differences on days 3 and 5. Although studies have described increased rates of complications, especially infectious complications, in association with postoperative hyperglycaemia, Cihoric *et al*.^4^ did not show any significant difference in infections between the groups. However, paradoxically, the incidence of septic shock requiring ICU admission was significantly lower in the dexamethasone group. Cihoric *et al*.^4^ have not specified whether participants received insulin to treat postoperative hyperglycaemia and that information would have been useful. Perhaps the reduction in inflammation produced by dexamethasone negated the adverse effects of hyperglycaemia. In addition, as the incidence of postoperative delirium was similar in both groups, administration of a single infusion of high-dose dexamethasone did not seem to produce neuropsychiatric adverse events in the short-term.

The trial by Cihoric *et al*.^4^ represents a major advance in perioperative care that has the potential to improve outcomes. However, it must be emphasized that it was powered to detect differences in the primary endpoint of inflammation, as quantified by CRP concentrations, and not to detect differences in the secondary endpoints. Larger trials may help unravel potential adverse events that a small trial may not detect. In addition, a platform design may help determine the optimal dose of dexamethasone. Treatment of postoperative hyperglycaemia with insulin may help improve results and also determine whether dexamethasone would be safe in people being treated with insulin for diabetes.
